# *Xyleborus bispinatus* Reared on Artificial Media in the Presence or Absence of the Laurel Wilt Pathogen (*Raffaelea lauricola*)

**DOI:** 10.3390/insects9010030

**Published:** 2018-02-28

**Authors:** Octavio Menocal, Luisa F. Cruz, Paul E. Kendra, Jonathan H. Crane, Miriam F. Cooperband, Randy C. Ploetz, Daniel Carrillo

**Affiliations:** 1 Tropical Research & Education Center, University of Florida 18905 SW 280th St, Homestead, FL 33031, USA; luisafcruz@ufl.edu (L.F.C.); jhcr@ufl.edu (J.H.C.); kelly12@ufl.edu (R.C.P.); dancar@ufl.edu (D.C.); 2 Subtropical Horticulture Research Station, USDA-ARS, 13601 Old Cutler Rd., Miami, FL 33158, USA; Paul.Kendra@ars.usda.gov; 3 Otis Laboratory, USDA-APHIS-PPQ-CPHST, 1398 W. Truck Road, Buzzards Bay, MA 02542, USA; miriam.f.cooperband@aphis.usda.gov

**Keywords:** ambrosia beetle, ambrosia fungi, beetle–fungus symbiosis, Coleoptera, Curculionidae, Lauraceae, *Persea americana*, *Persea humilis*, Scolytinae, Xyleborini

## Abstract

Like other members of the tribe Xyleborini, *Xyleborus bispinatus* Eichhoff can cause economic damage in the Neotropics. *X. bispinatus* has been found to acquire the laurel wilt pathogen *Raffaelea lauricola* (T. C. Harr., Fraedrich & Aghayeva) when breeding in a host affected by the pathogen. Its role as a potential vector of *R. lauricola* is under investigation. The main objective of this study was to evaluate three artificial media, containing sawdust of avocado (*Persea americana* Mill.) and silkbay (*Persea humilis* Nash.), for rearing *X. bispinatus* under laboratory conditions. In addition, the media were inoculated with *R. lauricola* to evaluate its effect on the biology of *X. bispinatus*. There was a significant interaction between sawdust species and *R. lauricola* for all media. Two of the media supported the prolific reproduction of *X. bispinatus*, but the avocado-based medium was generally more effective than the silkbay-based medium, regardless whether or not it was inoculated with *R. lauricola*. *R. lauricola* had a neutral or positive effect on beetle reproduction. The pathogen was frequently recovered from beetle galleries, but only from a few individuals which were reared on inoculated media, and showed limited colonization of the beetle’s mycangia. Two media with lower water content were most effective for rearing *X. bispinatus*.

## 1. Introduction

*Raffaelea lauricola* (T. C. Harr., Fraedrich & Aghayeva; Ophiostomatales: Ophiostomataceae) is a fungal pathogen carried primarily by the redbay ambrosia *Xyleborus glabratus* Eichhoff (Coleoptera: Curculionidae: Scolytinae) [1,2]. The pathogen is responsible for the vascular disease known as laurel wilt (LW), which affects American members of the Lauraceae family including redbay (*Persea borbonia* (L.) Spreng), swampbay (*Persea palustris* (Raf.) Sarg), silkbay (*Persea humilis* Nash), sassafras (*Sassafras albidum* (Nutall) Nees), pondspice (*Litsea aestivalis* (L.) Fernald), pondberry (*Lindera melissifolia* (Walter) Blume), and avocado (*Persea americana* Mill.) [1,3,4,5,6].

*X. glabratus* is rarely associated with LW-affected avocado trees in commercial plantings in south Florida [7,8]. Previously, the lateral transfer of *R. lauricola* to at least nine of the ambrosia beetle species, in addition to *X. glabratus*, was demonstrated in Florida [9,10]. Moreover, at least two of these species, *Xyleborus volvulus* (F.) and *Xyleborus ferrugineus* (F.), were shown to experimentally transmit the pathogen to avocado and redbay [9].

**Figure 1:**
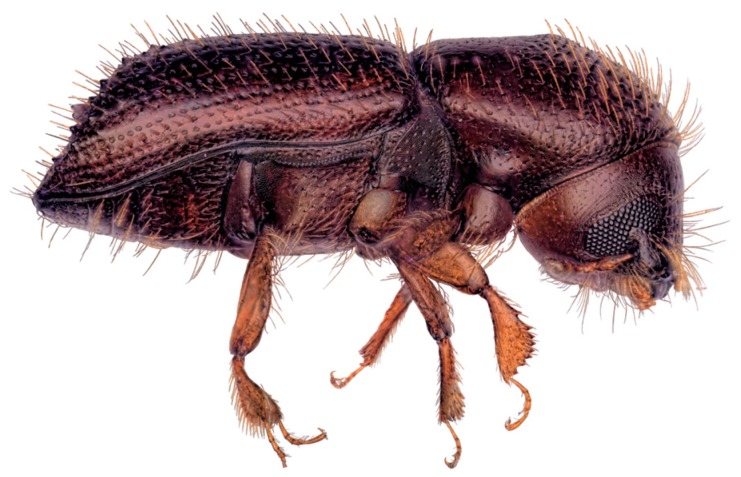
Adult female of *Xyleborus bispinatus* Eichhoff (lateral view).

*Xyleborus bispinatus* (Figure 1) was reported in Florida for the first time by Atkinson et al. [11]. Previously, it had not been distinguished from *X. ferrugineus*, a smaller, morphologically similar ambrosia beetle. Both species have broad distributions throughout South America, Central America, and in the southeastern United States [12,13], and *X. bispinatus* has also been reported in Italy [14].

Rabaglia et al. [15] stated that *X. ferrugineus* caused economic damage in lowland areas of the Neotropics. However, there are no supporting data for this statement. In forest ecosystems of central and southern Florida, *X. ferrugineus* and *X. bispinatus* occur sympatrically with *X. glabratus*, with which they reproduce in LW-affected swampbay and silkbay [16,17]. Recently, it has been shown shown that females of *X. bispinatus* incorporate *R. lauricola* in their mycangia [10], the storage organs for spores of symbiotic fungi [18]. Although the latter were not distinguished in *X. ferrugineus* by Carrillo et al. [9], it seems probable that the morphologically similar *X. bispinatus* can also transmit *R. lauricola* to avocado.

Introduced pathogens have had significant detrimental effects on forest communities. Relevant examples include *Cryphonectria parasitica* (Murril) M. E. Barr, the causal agent of chestnut blight [19], *Cronartium ribicola* Fisch, the causal agent of white pine blister rust [20], and *Ophiostoma novo-ulmi* Brasier, the pathogen that causes the Dutch elm disease [21]. For an increasing number of these diseases, scolytine beetles are vectors of the causal agents [22]. In general, there is limited information on the behavior and biology of these insects. However, with the help of artificial media, it may be possible to improve our understanding of their interactions with host plants and pathogens, as well as their roles in the epidemiology of these diseases.

The first ambrosia beetle species reared on an artificial medium was *X. ferrugineus* [23]. Since then, agar- and sawdust-based media have been used to rear and study the biology of several other ambrosia beetles [24,25,26,27,28].

Here, we describe a series of studies in which artificial media were evaluated for rearing *X. bispinatus*. The tested media incorporated sawdust from avocado or silkbay and were used to conduct preliminary studies on *X. bispinatus* and its biological response to *R. lauricola*.

## 2. Materials and Methods

### 2.1. Artificial Media

Sawdust from two hosts of *X. bispinatus* were evaluated. Logs of avocado were collected in February 2016 from an unsprayed avocado orchard in Miami-Dade County, FL (25°29′38″ N; 80°28′53″ W), and logs of silkbay were collected in February 2016 from the Archbold Biological Station in Highlands County, FL (27°10′50″ N; 81°21′0″ W). The logs were only used when they were found to be free of beetle infestation. Sawdust was produced as described by Castrillo et al. [29], and the rearing media were prepared as described by Menocal et al. [28]. 

Three types of media were evaluated (Table 1). Medium 1 contained either avocado or silkbay sawdust (hereafter, AM1 and SM1, respectively), as described by Castrillo et al. [29]. The ingredients of Medium 2 and Medium 3 (AM2; SM2 and AM3; SM3, respectively) were described by Biedermann et al. [30], but Medium 3 contained 120% more water than Medium 2.

### 2.2. Inoculation of Media

Twelve tubes of each medium were inoculated with an isolate of *R. lauricola* obtained from *X. glabratus* and identified as described by Dreaden et al. [31]. These tubes, denoted + RL, each received 8.2 × 10^6^ colony-forming units of the pathogen and were incubated at 25 °C for 10 days. 

### 2.3. Rearing Conditions and Dissection of Colonies

**Figure 2:**
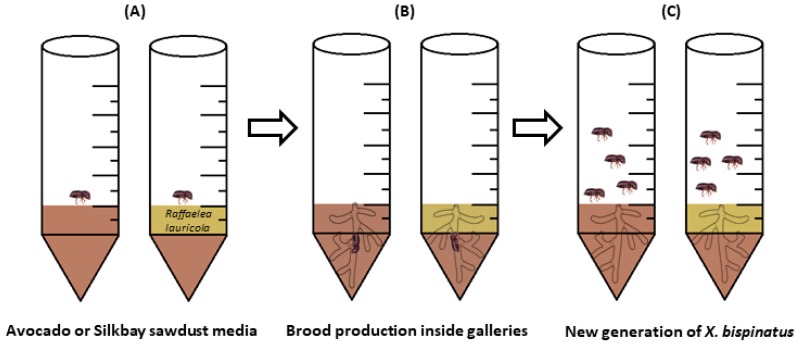
Schematic representation of the methodology used to rear *X. bispinatus*. (**A**) *X. bispinatus* females introduced into rearing tubes, (**B**) 25 days after female introduction), (**C**) 40 days after female introduction. A detailed description of the rearing methods is presented by Menocal et al. [28].

The rearing conditions were described by Menocal et al. [28]. Briefly, 12 active and fully sclerotized *X. bispinatus* females were selected from several healthy laboratory stock colonies, originally collected from LW-affected avocado trees. Individual females were dipped in 70% ethanol for 5 s to reduce external contaminants and then individually placed into rearing tubes (Figure 2) and incubated in complete darkness at 25 ± 1 °C and 75% RH (Relative humidity). The surface of the medium and sides of the tubes were inspected every 2–3 days, and whenever galleries, eggs, larvae, pupae, and adults were first perceived, these observations were recorded (Figure 2).

Forty days after female introduction, beetle colonies were destructively sampled in a laminar-flow hood. Different life stages were quantified: first by observing the surface and the sides of the tubes and then by emptying the colony contents into a Petri dish, as described by Cooperband et al. [27]. Eggs, larvae, pupae, and adults were gently removed from the medium and quantified. Motionless adults were recorded as dead, and the foundress female was not tallied. To produce a second generation, 12 fully sclerotized females from each treatment were surface-disinfested for 5 s in 70% ethanol and reared as above with the same medium and treatment. The experiment concluded after the emergence of the second generation of females.

### 2.4. Fungal Isolation and Identification

From each − and + RL first- and second-generation tube, one fully sclerotized female was assayed for *R. lauricola*. Females were surface-disinfested with 70% ethanol for 30 s and rinsed three times with sterile deionized water; the heads and bodies were macerated separately in 200 µL of sterile water using a motorized tissue grinder (Fisher Scientific Catalog No. 12141361); 100 µL of macerate from each body part was then streaked on 9-cm-diameter plates of CSMA medium (cycloheximide, streptomycin, malt and agar), as described by Harrington et al. [32]. The gallery surfaces were sampled with a sterile inoculation loop (Fisher Scientific Catalog No. 22170206) and streaked on CSMA. After 7 days, the colonies that phenotypically resembled the pathogen were quantified and confirmed as *R. lauricola* with the diagnostic microsatellite markers CHK and IFW of Dreaden et al. [31]. In addition, other fungi which were isolated from the other 12 females and galleries were identified by amplifying a portion of the nuclear large subunit 28S ribosomal DNA, as described by Menocal et al. [28].

### 2.5. Data Analysis

Data were analyzed with two-way analyses of variance (PROC GLIMMIX, SAS v9.3, SAS Institute 2010, Cary, NC, USA). Each medium was evaluated independently and considered a separate experiment. Statistical interactions between sawdust source and − and + inoculation with *R. lauricola* were assessed. Since the data from the first and second generations were similar, they were pooled for the analysis. Data were square root-transformed before analysis to meet the normality assumption. Means were separated statistically with Tukey’s HSD.

## 3. Results

### 3.1. Medium 1

Foundress females established first and second generations in all tubes. Eggs were not observed in galleries along the rearing tube walls. However, in AM1 + RL, larvae, pupae, and new adults began to be observed at 12, 15, and 21 days, respectively, after female introduction. In AM1, larvae, pupae, and new adults were observed at 15, 18, and 24 days after female introduction. In SM1 + RL, larvae, pupae, and new adults were observed at 18, 23, and 29 days after female introduction, and in SM1, larvae, pupae, and new adults were observed at 15, 21, and 27.

**Figure 3:**
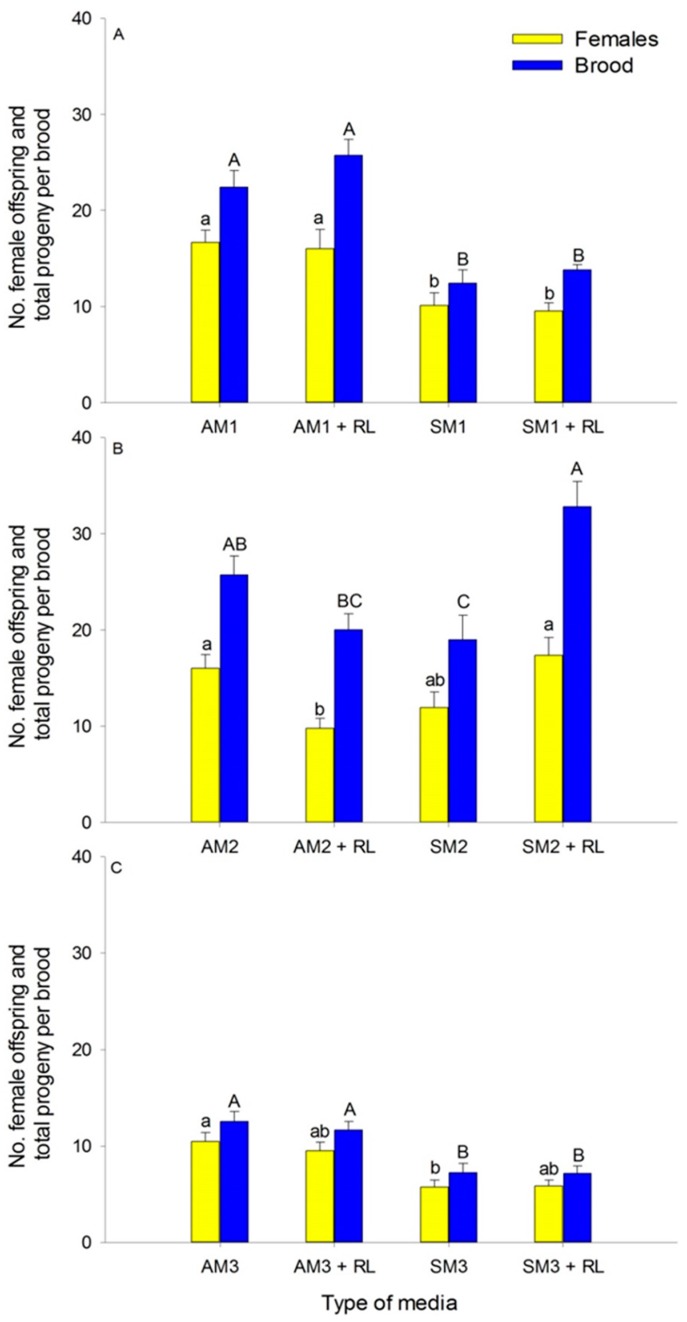
Number of *X.s bispinatus* female offspring and total brood (including females) produced by one foundress female. Artificial media were (**A**) (medium 1), (**B**) (medium 2), and (**C**) (medium 3), prepared with either avocado (AM) or silkbay (SM) sawdust, and either inoculated (+ RL) or not with *Raffaelea lauricola*. Bars represent mean numbers of female offspring (yellow) and total brood (blue) produced per foundress ± SE. For a given medium, offspring and total brood with the same letters are not significantly different (Tukey’s HSD test: *p* < 0.05).

The interaction between sawdust species and *R. lauricola* was significant (*F*_3,95_ = 18.60, *p* < 0.0001); total brood and adult female offspring were significantly greater on media containing avocado sawdust (either with or without *R. lauricola*) than on media containing silkbay sawdust (Table 2, Figure 3A). The number of males per brood ranged from zero to four. Males were present in 91% of AM1 + RL, 88% of AM1, 79% of SM1 + RL, and 58% of SM1. Adult mortality in the colonies was low in all treatments and decreased in the following order: AM1 (7%), AM1 + RL (6%), SM1 (5%), SM1 + RL (3%).

### 3.2. Medium 2

The percentage of foundress females that established colonies in SM2 + RL, AM2, and SM2, decreased from 100 to 96 from the first to the second generation. In AM2 + RL, foundress females established 100% of colonies for both generations. During tunnel inspections, no eggs were visible along the galleries through the rearing tube walls. Larvae, pupae, and new adults were observed in galleries visible through the tubes at 12, 16, and 21 days, respectively, after a female founder had been introduced into SM2 + RL. In AM2, larvae, pupae, and new adults were observed at 16, 20, and 25 days after female introduction. In the case of AM2 + RL, larvae, pupae, and new adults were observed at 18, 23, and 29 after female introduction. Finally, in SM2, larvae, pupae, and new adults were visible at 16, 21, and 25 days after the female foundress was introduced.

There was a significant interaction between sawdust species and the presence of *R. lauricola* (*F*_3,95_ = 13.41, *p* < 0.0001) (Table 3, Figure 3B). In the avocado sawdust, adult female offspring (but not the total brood) was significantly different in inoculated and not inoculated media. By contrast, in the silkbay sawdust, total brood (but not the adult female offspring) greatly increased in the presence of *R. lauricola* (Table 3, Figure 3B). The number of males per brood ranged from zero to two. Males were seen in 88% of the colonies in SM2 + RL, 88% of AM2, 83% of AM2 + RL, and 71% of SM2. As with medium 1, adult mortality was low for all treatments: AM2 (6.6%), AM2 + RL (5.1%), SM2 + RL (3.6%), and SM2 (2.9%).

### 3.3. Medium 3

The percentage of foundress females that established colonies in AM3 was 100 and 83 in the first and second generations, respectively. In AM3 + RL, 100% of females established colonies in both generations. In SM3, 85% of females established colonies in both generations. When using SM3 + RL, the percentage of colony establishment decreased from 100% to 91%. During gallery inspections, as was the case with medium 1 and 2, no eggs were visible in galleries along the rearing tubes walls. Larvae, pupae, and new adults were first observed at 17, 21, and 25 days, respectively, after the introduction of the female foundress in AM3. In AM3 + RL, larvae, pupae, and new adults were first observed 19, 22, and 27 days after female introduction. In SM3, larvae, pupae, and new adults were seen at 20, 25, 30 days, and in SM3 + RL at 18, 24, and 28 days after female introduction.

The interaction effect between *R. lauricola* and sawdust species was significant (*F*_3,95_ = 7.57, *p* = 0.0001); brood production was significantly greater on avocado sawdust (regardless of inoculation) than silkbay sawdust, with or without *R. lauricola* (Table 4, Figure 3C). Adult female offspring was greater on avocado sawdust than on silkbay sawdust that was not inoculated with *R. lauricola*, but there was no difference when the media of both sawdust species were inoculated with *R. lauricola* (Table 4, Figure 3C). The number of males per colony ranged from zero to three. Males were present in 79% of the colonies in AM3, 66% in AM3 + RL, 70% in SM3, and 66% and SM3 + RL. Adult mortality was similar in AM3 (5.2%), SM3 (7.8%), and SM3 + RL (4.5%). By contrast, mortality was greater in AM3 + RL (12.9%).

### 3.4. Recovery of R. lauricola and Other Fungi from X. bispinatus Reared on Artificial Media

Adult females reared on + RL carried a low number of colony forming units (CFUs) of *R. lauricola* (Table 5). *R. lauricola* was recovered more frequently from females reared on silkbay medium than on avocado-based media (Table 5). Typically, *R. lauricola* was recovered more frequently from the heads than from the bodies (Table 5). The fungus was recovered from 11 out of 24 beetle galleries in AM1 + RL, and from 22 of 24 beetle galleries in SM1 + RL. Similarly, *R. lauricola* was recovered from 14 of 24 beetle galleries in AM2 + RL, and 15 of 24 beetle galleries in SM2 + RL. Finally, the fungus colonized 14 of 24 beetle galleries in AM23 + RL, and 21 of 24 beetle galleries in SM3 + RL.

On CSMA, other fungi besides *R. lauricola* were recovered from the heads, bodies, and galleries of *X. bispinatus* reared in avocado and silkbay media (Table 6). Five other species were isolated from colonies inoculated with *R. lauricola*, and six species from colonies not inoculated with *R. lauricola*. *Raffaelea subalba* (T. C. Harr., Aghayeva & Fraedrich) was the most abundant and most frequently found fungus, recovered from beetles’ heads and bodies in both avocado and silkbay media (Table 6). However, in galleries, the most frequent fungus was *Raffaelea subfusca* (T. C. Harr., Aghayeva & Fraedrich) (Table 6). *Raffaelea arxii* T. C. Harr., Aghayeva & Fraedrich was isolated at a low frequency and abundance. *Candida multigemmis* (Buhagiar) S. A. Mey & Yarrow (Saccharomycetales) was isolated from all treatments and only from beetles’ heads. *Alloascoidea* sp. (Saccharomycetales: Alloascideaceae) and *Phaeoacremonium inflatipes* W. Gams, Crous & M. J. Wingf (Diaporthales: Togniniaceae) were only isolated from beetle galleries (Table 6).

## 4. Discussion

Interactions between plants and insects have promoted insect diversity, but, in many cases, additional factors have enhanced diversification. For example, insect–plant interactions often include microbial associates, some of which are fungal pathogens [22,33]. Ambrosia beetles rely on fungal symbionts to fulfill their nutritional requirements [34,35]. However, the associations among symbiotic fungi and ambrosia beetles remain severely understudied.

The reproduction and survival of ambrosia beetles depends on the growth and quality of fungal symbionts in the colony [36]. Fungal growth, and therefore the reproductive potential of *X. bispinatus* in this study, may have been influenced by the amount of sucrose, casein, yeast, starch, and water in the media. Robinson et al. [37] found that the sugar content in wood is a significant factor determining fungal colonization by *Ophiostoma piceae* Munch (Ophiostomatales: Ophiostomataceae). In addition, Abraham et al. [38] found that nitrogen availability affected the growth of *O. piceae*. Like most *Ophiostoma* species, *O. piceae* utilizes ammonium, but not nitrate, as a nitrogen source [39]. Nitrogen is typically scarce in wood [40,41], ranging between 0.01 and 0.1% of the dry weight. Other ingredients in artificial media, such as yeast and starch, have been reported to be important for ambrosia beetle reproduction. Mizuno & Kajimura [42] reported more progeny of *Xyleborus pfeili* (Ratzeburg) in media containing five times more yeast and starch. In this study, reproduction of *X. bispinatus* was greatest on a medium that contained the most yeast and starch (Medium 1) (Table 1). However, Maner et al. [26] stated that increasing levels of yeast and starch (up to 4 times) did not enhance the reproduction of *X. glabratus*. Interestingly, female mortality increased in the medium with greater levels of these nutrients, yet, when females survived, they produced more brood [26]. Overall, this and other studies suggest that ambrosia beetles (depending on the species) require different amounts of yeast and starch in the rearing media, but that an excess of these substances might result in reduced fitness. Similarly, the amount of water in the media appeared to impact the growth of the symbionts, therefore affecting the beetles’ fitness. Although we did not compare the reproduction between the Medium 2 and Medium 3 in the present study, females of *X. bispinatus* produced fewer progeny in the medium with higher water content (Figure 3C).

In this study, several symbiotic fungi were found associated with *X. bispinatus*. Three *R.* species (*R. arxii*, *R. subalba*, *R. subfusca*) were frequently associated with galleries, heads, and bodies, suggesting that they are nutritional symbionts of *X. bispinatus*. Recently, Saucedo et al. [43] has demonstrated that production of male progeny by females of *X. bispinatus* is possible when these symbionts are individually offered in artificial media amended with these fungi. Moreover, the indiscriminate association of *X. bispinatus* with these three species suggests that this beetle may not have an obligatory relationship with one specific fungus to fulfill its nutritional requirements, as observed for *Dendroctonus brevicomis* LeConte [44]. Additional experiments are needed to evaluate the effect of varying amounts of the rearing media’s main ingredient on these fungi. Currently, in order to rear *X. bispinatus*, we recommend Medium 1 with avocado sawdust, as described by Castrillo et al. [25]. This medium would yield more female adults. In addition, rearing *X. bispinatus* in the presence of *R. lauricola* or other *Raffaelea* species could facilitate future beetle–fungal symbiosis research.

Previously, it has been documented that other ambrosia beetles (including *X. bispinatus*) carry *R. lauricola* [10], the primary nutritional symbiont of *X. glabratus* [2,26], in their mandibular mycangia. In our experiments, the mycangia of *X. bispinatus* were scarcely colonized by *R. lauricola*, whereas other *Raffaelea* species were more frequent and abundant. Our results differ from those of Saucedo et al. [43], in that they found higher numbers of CFUs of the pathogen in *X. bispinatus* mycangia.

It could be hypothesized that the colonization of the mycangia of any ambrosia beetle is influenced by the first fungi it encounters at the moment when pupae (which do not yet possess mycangia) develop into adults. As the female adults that were employed to establish colonies in the present study did possess mycangia, which were presumably pre-colonized with other symbionts, it is possible that there were reduced opportunities for *R. lauricola* to colonize their mycangia, compared to those in the study of Saucedo et al. [43], in which colonies of *X. bispinatus* were established with individuals that were exposed to a single symbiont. In the latter situation, it seems probable that there would be an increased opportunity for colonization by *R. lauricola,* especially when new adults of *X. bispinatus* had only been exposed to that fungus. Clearly, a better understanding of how ambrosia beetles establish and maintain an association with specific symbionts is needed [45].

In our study, the sawdust of two host tree species affected the development and reproduction of *X. bispinatus*. These results are comparable to other studies where ambrosia beetles were artificially reared in different types of sawdust [25]. There is also evidence of differences in host suitability for ambrosia beetles using field collected logs as the rearing substrate. Brar et al. [46] reared *X. glabratus* on avocado, redbay, and swampbay logs. They found that swampbay was a better host for *X. glabratus* than the other two species. The results of our study suggest that avocado is a better host for *X. bispinatus* than silkbay. Since we obtained more progeny on artificial media than Brar et al. [46], artificial media may be a superior method for establishing colonies of ambrosia beetles in laboratory conditions, as opposed to logs.

Foundress females initiated tunneling activities almost immediately after they were introduced into the rearing tubes. Active tunneling was evident by the appearance of copious frass pushed out of the gallery through the entry hole. Frass production continued throughout the life cycle of the beetle and was a sign of colony health. Approximately five days after female introduction, the first galleries were observed along the rearing tube walls, together with fungal growth on the surface of the rearing media. Two weeks after female introduction, the first immature stages were observed in visible galleries or on the surface of the rearing media. The first new adult females were observed approximately 3 weeks after female introduction. These adults remained inside the galleries for approximately 1 week, probably mating with sibling males and helping in colony maintenance [46]. By the fourth week after female introduction, adults started to emerge from the rearing medium and sometimes exhibited an aggressive behavior, chewing underneath the lid or through the plastic walls of the tube. This behavior was typical after 35 days of female introduction, once the colonies were crowded and could be related to their natural instinct to disperse at that stage. At the time of dissection (day 40), new adults and the original female were found alongside other developmental stages; new adults were dark brown and very active, whereas the foundress female was typically black and scarcely moving.

The performance of *X. bispinatus* was similar to other ambrosia beetles that were previously reared on artificial media [24,25,26,27]. In our study, few females, likely unmated, produced offspring composed of males only; this observation suggests that, like other ambrosia beetles, *X. bispinatus* possess haplodiploid reproduction: males are haploid derived from unfertilized eggs, and females are diploid derived from fertilized eggs [47]. However, approximately 5% of broods did not contain males during two consecutive generations. It is unclear whether the females did not produce males at all or whether they died prematurely and were degraded before being tallied. However, male-less broods have also been reported in other ambrosia beetles including *Xylossandrus compactus* (Eichh.) [48], *Xylosandrus germanus* (Blandford) [49], *Xyleborinus saxesenii* (Ratzeburg) [50], and *Euwallacea* spp. [27].

*X. bispinatus*, like other ambrosia beetles, also exhibits female-biased sex ratios as a consequence of local mate competition (LMC) [51]. Hamilton [51] indicated that the following principles define LMC: the females greatly outnumber the males, development is gregarious, reproduction is arrhenotokous, males are disinclined to emigrate from the colony, every colony has at least one male, males hatch sooner than females and can mate multiple times, mating occurs soon after female eclosion, and females can store sperm after mating. The results of this study verify that *X. bispinatus* meets the first three principles of LMC. However, the fourth principle (one male in every brood) was not met, and the remaining four principles could not be confirmed.

The methods developed by Menocal et al. [28] and tested here may facilitate the study of LMC principles and other aspects of the biology of *X. bispinatus*, such as the interactions with symbiotic fungi. Moreover, laboratory colonies could be used to understand processes that influence *R. lauricola* acquisition by *X. bispinatus* and the persistence of this association. Laboratory rearing of *X. bispinatus* on artificial media may greatly facilitate behavioral, physiological, and ecological studies of this important ambrosia beetle.

## Figures and Tables

**Table 1:** Three experiments were conducted using ingredients of three artificial media for rearing *X. bispinatus*, using either avocado or silkbay sawdust.

Ingredients	Media			Manufacturer/Source
	Type 1 AM1 or SM1	Type 2 AM2 or SM2	Type 3 AM3 or SM3	
Sawdust	45 g	84 g	84 g	Avocado or silkbay wood
Granulated agar	12 g	12.6 g	12.6 g	Difco Agar, Dickinson & Company, Sparks, MD, USA
Sucrose	6 g	2.1 g	2.1 g	Fisher Scientific, Fair Lawn, NJ, USA
Starch	3 g	2.1 g	2.1 g	Fisher Science Education, Nazareth, PA, USA
Yeast	3 g	2.1 g	2.1 g	Fisher Science Education, Nazareth, PA, USA
Casein	3 g	4.2 g	4.2 g	MP Biomedicals, LLC, Solon, OH, USA
Wesson’s salt mixture	0.6 g	0.52 g	0.52 g	MP Biomedicals, LLC, Solon, OH, USA
Tetracycline	0.21 g	0.14 g	0.14 g	Fisher Scientific, Fair Lawn, NJ, USA
Wheat germ oil	1.5 mL	1.05 mL	1.05 mL	Frontier Scientific Services, Newark, DE, USA
Peanut oil	-	1.05 mL	1.05 mL	Ventura Foods, LLC, Brea, CA, USA
95% ethanol	3 mL	2.1 mL	2.1 mL	Decon Labs, Inc., King of Prussia, PA, USA
Distilled H_2_O	370 mL	244 mL	540 mL	

Type 1 medium from Castrillo et al. [29], using avocado or silkbay sawdust; Type 2 medium from Biedermann et al. [30], using avocado or silkbay sawdust; Type 3 medium from Biedermann et al. [30], using avocado or silkbay sawdust modified with extra water. AM = Avocado medium; SM = Silkbay medium.

**Table 2:** Developmental stages and biological parameters of *X. bispinatus* on medium 1 based on either avocado or silkbay sawdust, either inoculated or uninoculated with *R. lauricola* (*N* = 24).

Medium	Mean ± SE of Offspring per Tube after 40 Days						% of Females per Colony	*N* with Offspring (Any Stage) (%)	*N* with Females ^1^ (%)
	Eggs	Larvae	Pupae	Male Adults	Female Adults	Brood (All Stages Combined)			
AM1	0.00 ± 0.00	4.08 ± 0.82	0.71 ± 0.26	0.96 ± 0.11	16.67 ± 1.27	22.42 ± 1.72	74%	24 (100%)	24 (100%)
AM1 + RL	0.38 ± 0.24	5.17 ± 0.96	2.63 ± 0.69	1.58 ± 0.31	16.00 ± 2.00	25.75 ± 1.65	62%	24 (100%)	19 (79%)
SM1	0.17 ± 0.17	1.21 ± 0.32	0.25 ± 0.09	0.67 ± 0.14	10.13 ± 1.29	12.42 ± 1.39	82%	24 (100%)	24 (100%)
SM1 + RL	0.21 ± 0.15	2.42 ± 0.60	0.58 ± 0.19	1.08 ± 0.18	9.54 ± 0.86	13.83 ± 0.53	69%	24 (100%)	21 (88%)

AM1 = Avocado medium 1; AM1 + RL = Avocado medium 1 inoculated with *R. lauricola;* SM1 = Silkbay medium 1; SM1 + RL = Silkbay medium 1 inoculated with *R. lauricola. N* = number of rearing tubes used per treatment. ^1^ Number of rearing tubes with at least one female offspring.

**Table 3:** Developmental stages and biological parameters of *X. bispinatus* on medium 2, based on either avocado or silkbay sawdust, either inoculated or uninoculated with *R. lauricola* (*N* = 24).

Medium	Mean ± SE of Offspring per Tube after 40 Days						% of Females per Colony	*N* with Offspring (Any Stage) (%)	*N* with Females ^1^ (%)
	Eggs	Larvae	Pupae	Male Adults	Female Adults	Brood (All Stages Combined)			
AM2	1.08 ± 0.36	6.00 ± 0.99	1.63 ± 0.31	1.00 ± 0.17	16.00 ± 1.42	25.71 ± 1.94	62%	23 (96%)	23 (96%)
AM2 + RL	1.50 ± 0.59	6.21 ± 0.66	1.58 ± 0.24	0.96 ± 0.11	9.75 ± 1.03	20.00 ± 1.65	49%	24 (100%)	21 (88%)
SM2	0.29 ± 0.22	4.79 ± 0.88	1.13 ± 0.26	0.83 ± 0.13	11.92 ± 1.61	18.96 ± 2.56	63%	23 (96%)	22 (92%)
SM2 + RL	2.58 ± 0.67	8.63 ± 1.21	3.21 ± 0.34	1.04 ± 0.11	17.33 ± 1.85	32.79 ± 2.62	53%	23 (96%)	22 (92%)

AM2 = Avocado medium 2; AM2 + RL = Avocado medium 2 inoculated with *R. lauricola;* SM2 = Silkbay medium 2; SM2 + RL = Silkbay medium 2 inoculated with *R. lauricola. N* = number of rearing tubes used per treatment. ^1^ Number of rearing tubes with at least one female offspring.

**Table 4:** Developmental stages and biological parameters of *X.bispinatus* on medium 3, based on either avocado or silkbay sawdust, either inoculated or uninoculated with *R. lauricola* (*N* = 24).

Medium	Mean ± SE of Offspring per Tube after 40 Days						% of Females per Colony	*N* with Offspring (Any Stage) (%)	*N* with Females ^1^ (%)
	Eggs	Larvae	Pupae	Male Adults	Female Adults	Brood (All Stages Combined)			
AM3	0.08 ± 0.05	1.21 ± 0.27	0.00 ± 0.00	0.79 ± 0.08	10.46 ± 0.91	12.54 ± 1.03	83%	22 (92%)	22 (92%)
AM3 + RL	0.00 ± 0.00	1.13 ± 0.26	0.17 ± 0.10	0.83 ± 0.15	9.50 ± 0.86	11.63 ± 0.89	82%	24 (100%)	23 (96%)
SM3	0.04 ± 0.04	0.58 ± 0.30	0.17 ± 0.11	0.71 ± 0.09	5.71 ± 0.74	7.21 ± 0.99	79%	20 (83%)	20 (83%)
SM3 + RL	0.00 ± 0.00	0.42 ± 0.21	0.21 ± 0.10	0.67 ± 0.10	5.83 ± 0.63	7.13 ± 0.79	82%	23 (96%)	23 (96%)

AM3 = Avocado medium 3; AM3 + RL = Avocado medium 3 inoculated with *R. lauricola;* SM3 = Silkbay medium 3; SM3 + RL = Silkbay medium 3 inoculated with *R. lauricola. N* = number of rearing tubes used per treatment. ^1^ Number of rearing tubes with at least one female offspring.

**Table 5:** Recovery and frequency of *R. lauricola* from females of *X. bispinatus* reared on artificial media previously inoculated with this fungus.

Medium Type ^1^	Host	Mean No. of CFUs ± SE per Head & Pronotum	Frequency *(n/N)*	Mean No. of CFUs ± SE per Body Lacking Head & Pronotum	Frequency *(n/N)*
Medium 1	Avocado	18.9 ± 8.9	7/24	5.3 ± 3.3	3/24
	Silkbay	9.7 ± 3.0	6/24	84	1/24
Medium 2	Avocado	6.7 ± 2.5	6/24	20	1/24
	Silkbay	28.9 ± 10.3	10/24	2	1/24
Medium 3	Avocado	5.4 ± 2.9	5/24	2	1/24
	Silkbay	6 ± 1.0	5/24	2	1/24

*n*: number of individuals testing positive for the presence of *R. lauricola*; *N*: Number of individuals tested; CFUs: colony-forming units of *R. lauricola*. ^1^ Note that each medium was evaluated separately and was considered a separate experiment.

**Table 6:** Fungal species isolated from 12 *X. bispinatus* beetles and their galleries collected from either avocado- or silkbay-based media. Fungi were isolated from the head and pronotum or from the body, when head and pronotum were lacking.

Treatments	Species	Medium Containing Avocado Sawdust					Medium Containing Silkbay Sawdust				
		Head and Pronotum		Body ^a^		Gallery	Head and Pronotum		Body ^a^		Gallery
		Avg. CFU/Beetle	Freq. *n/N*	Avg. CFU/Beetle	Freq. *n/N*	Freq. *n/N*	Avg. CFU/Beetle	Freq. *n/N*	Avg. CFU/Beetle	Freq. *n/N*	Freq. *n/N*
Media inoculated with *R. lauricola*	*Raffaelea arxii*	125	10/12	0	0/12	0/12	70.2	7/12	0	0/12	0/12
	*Raffaelea lauricola*	4.67	3/12	0	0/12	8/12	23.8	6/12	23	1/12	6/12
	*Raffaelea subalba*	474.5	11/12	0	0/12	0/12	229	11/12	117	9/12	10/12
	*Raffaelea subfusca*	0	0/12	0	0/12	9/12	0	0/12	0	0/12	0/12
	*Phaeoacremonium inflatipes*	0	0/12	0	0/12	4/12	0	0/12	0	0/12	2/12
	*Candida multigemmis*	9.1	9/12	0	0/12	0/12	80.1	9/12	0	0/12	0/12
Media non-inoculated with *R. lauricola*	*Raffaelea arxii*	40.5	9/12	0	0/12	0/12	42	3/12	0	0/12	0/12
	*Raffaelea lauricola*	0	0/12	0	0/12	0/12	0	0/12	0	0/12	0/12
	*Raffaelea subalba*	199.7	11/12	0	0/12	0/12	253.1	12/12	0	0/12	0/12
	*Raffaelea subfusca*	35.2	12/12	0	0/12	10/12	0	0/12	0	0/12	11/12
	*Phaeoacremonium inflatipes*	0	0/12	0	0/12	0/12	0	0/12	0	0/12	4/12
	*Candida multigemmis*	37.3	10/12	0	0/12	0/12	29.7	10/12	0	0/12	0/12
	*Alloascoidea* sp.	0	0/12	0	0/12	6/12	0	0/12	0	0/12	0/12

**^a^** Body separated from the head and pronotum. Freq. = Frequency of fungal species detected; Avg. = Average; *n* = number of beetle body parts or number of galleries that were positive for a fungal species; *N* = Number of beetles or galleries tested for a specific fungus.
